# Field Assessment of a Novel Household-Based Water Filtration Device: A Randomised, Placebo-Controlled Trial in the Democratic Republic of Congo

**DOI:** 10.1371/journal.pone.0012613

**Published:** 2010-09-10

**Authors:** Sophie Boisson, Mbela Kiyombo, Larry Sthreshley, Saturnin Tumba, Jacques Makambo, Thomas Clasen

**Affiliations:** 1 Department of Infectious and Tropical Diseases, London School of Hygiene and Tropical Medicine, London, United Kingdom; 2 Ecole de Santé publique de Kinshasa, Kinshasa, République Démocratique du Congo; 3 Communauté Presbytérienne de Kinshasa, Kinshasa, République Démocratique du Congo; 4 Zone de Santé de Bibanga, Province du Kasaï Oriental, République Démocratique du Congo; Aga Khan University, Pakistan

## Abstract

**Background:**

Household water treatment can improve the microbiological quality of drinking water and may prevent diarrheal diseases. However, current methods of treating water at home have certain shortcomings, and there is evidence of bias in the reported health impact of the intervention in open trial designs.

**Methods and Findings:**

We undertook a randomised, double-blinded, placebo-controlled trial among 240 households (1,144 persons) in rural Democratic Republic of Congo to assess the field performance, use and effectiveness of a novel filtration device in preventing diarrhea. Households were followed up monthly for 12 months. Filters and placebos were monitored for longevity and for microbiological performance by comparing thermotolerant coliform (TTC) levels in influent and effluent water samples. Mean longitudinal prevalence of diarrhea was estimated among participants of all ages. Compliance was assessed through self-reported use and presence of water in the top vessel of the device at the time of visit. Over the 12-month follow-up period, data were collected for 11,236 person-weeks of observation (81.8% total possible). After adjusting for clustering within the household, the longitudinal prevalence ratio of diarrhoea was 0.85 (95% confidence interval: 0.61–1.20). The filters achieved a 2.98 log reduction in TTC levels while, for reasons that are unclear, the placebos achieved a 1.05 log reduction (p<0.0001). After 8 months, 68% of intervention households met the study's definition of current users, though most (73% of adults and 95% of children) also reported drinking untreated water the previous day. The filter maintained a constant flow rate over time, though 12.4% of filters were damaged during the course of the study.

**Conclusions:**

While the filter was effective in improving water quality, our results provide little evidence that it was protective against diarrhea. The moderate reduction observed nevertheless supports the need for larger studies that measure impact against a neutral placebo.

**Trial Registration:**

Current Controlled Trials ISRCTN03844341

## Introduction

Diarrhoea is responsible for 1.8 million deaths annually, mostly among children under five in developing countries[1]. Much of this disease burden is attributable to unsafe water, poor hygiene and sanitation[2]. An estimated 884 million people worldwide lack access to improved water sources[3]; hundreds of millions more rely on improved sources that are not consistently safe for drinking. Even water that is safe at the point of distribution often becomes contaminated during collection, transport and storage within the home due to poor hygiene conditions and practices[4].

While safe, reliable, piped-in water is an essential goal, treating water at the household or other point of consumption provides a means by which vulnerable populations can improve the quality of their own drinking water[5]. The practice is widespread, with hundreds of millions reporting that they usually treat their water at home before drinking it [6].

There is also evidence that household water treatment is protective against diarrhoea [7], [8], [9] though research suggests that placebo effect and reporting bias play a role in the estimate of effect reported in open trials[10], [11], [12], [13]. Placebo-controlled trials of chlorine-based interventions have been conducted [14], [15], but apart from a recent study in Ghana[16], none have assessed the neutrality of the placebo or the effectiveness of the blinding, and other issues have been raised about their methodological quality. Filters are more difficult to blind among populations relying on unimproved water. If the water is turbid, a placebo that contains no filter medium is readily identified by comparing its effluent with the effective filter. However, a placebo that removes turbidity to ensure blinding will probably also remove pathogens that tend to adhere to the suspended solids; it may also create adsorption sites or promote biofilm adhesion that will also render the “placebo” at least somewhat effective in removing pathogens. To date, the only placebo-controlled trials of household-based filters have been conducted in the United States with municipally treated water that is low in turbidity but also met WHO water quality standards[16], [17], [18], [19]. Thus, these results cannot be generalised to settings with turbid and contaminated water.

Several water treatment methods have been promoted in low-income settings, including disinfection, disinfection/flocculation, ceramic filtration, solar disinfection and boiling[5].Each has limitations in terms of microbiological effectiveness, cost, acceptability, environmental impact, and sustainability among target populations[20]. Moreover, except for boiling, none of these interventions have achieved scale except in limited settings [6]. This has led to calls for alternative technologies that are effective against the full array of microbial pathogens, that can be deployed and used at a large scale with minimum programmatic support, and that will be embraced by the target population[21].

The Lifestraw Family® is a newly developed household-based gravity filter that employs hollow-fibre membranes to remove waterborne pathogens by ultrafiltration. Independent laboratory testing has shown the device to meet the US Environmental Protection Agency (USEPA) standards for bacteria, viruses and protozoan cysts[22]. The device is designed to treat a minimum of 18,000 L of water and assumed to last for about three years. The manufacturer, Vestergaard-Frandsen SA of Lausanne, Switzerland, plans to sell the filter in large volumes for about US$20.

## Methods

### Study design; sample size calculation

The study was designed as a randomised, double-blinded, placebo-controlled trial. Our primary outcome was longitudinal prevalence of diarrhea defined as the number of weeks with diarrhea divided by the total number of weeks under observation. The study was powered to detect a 30% reduction in the mean longitudinal diarrhoea between the two groups. This was a conservative estimate in comparison with the pooled risk reduction of 63% calculated from six previous studies of household filters[8]. The calculation assumed 80% power, α = 0.05, a baseline longitudinal prevalence of diarrhoea of 5%, and a coefficient of variation of 2. In order to account for potential lost to follow-up (10%) as well as clustering of diarrhoea within household and intermittent surveillance (7-day period prevalence measured repeatedly once a month over the 12-month follow-up period) (10%), we estimated that we needed at least 600 individuals in each arm. Assuming a mean of 5 persons per household, the number of households to be recruited was approximately 120 per arm, or 240 households in total. The protocol for this trial (Protocol S1) and CONSORT checklist (Checklist S1) are available as supporting information.

### Setting and participant eligibility

The study was conducted from April 2008 to July 2009 in the rural health zone of Bibanga, 80 km from the city of Mbuji-Mayi in the eastern province of Kasai, the Democratic Republic of Congo (DRC). Despite abundant water resources, more than three quarters of the population in rural areas in the DRC rely on unimproved water sources for drinking, mainly surface water and unprotected springs[23]. With the assistance of the Presbyterian Church of Kinshasa, which has been supporting community health programmes in this area for many years, and staff at the health zone level, we identified possible study sites. Selected communities relied on unimproved water sources that tested over 1000 thermotolerant coliforms (TTC)/100 ml, reported low use of household water treatment, were easily accessible all year round from the reference hospital of Bibanga where the field team was established, and were motivated to take part in the project. In order to meet the sample size requirements, the study was conducted in two neighbouring villages.

### Intervention

Each intervention household received a Lifestraw Family filter and each control household received a placebo. The Lifestraw Family is a gravity-fed microbiological water purifier. Water is poured into a 2.5 L plastic vessel, passes through a 27-µm pre-filter, and flows down a 1 m long plastic pipe before passing through the filtration cartridge comprised of hollow-fibres with a 20-nm pore size. The top vessel contains a slow eluding chlorine tablet designed to prevent biofilm formation and increase the life of the cartridge. Treated water is accessed from the side of the cartridge via a tap. The device is cleaned daily by rinsing the pre-filter and backwashing the cartridge using a squeeze-pump and outlet valve mounted on the bottom of the cartridge. The device is designed to treat at least 18,000 L of water with a flow rate of approximately 150 ml per minute or 9 L per hour. In the laboratory, the filter was found to meet the USEPA standards for microbiological water purifiers by reducing bacteria by 6.9 logs, viruses by 4.7 logs and protozoan cysts by 3.6 logs [22].

### Placebo

The placebo had the same configuration, appearance and external components as the Lifestraw Family except that (i) the chlorine tablet was removed from the upper vessel to prevent possible microbicidal action, (ii) the filtration membranes were replaced by some extra piping to imitate the weight and effluent flow rate of the real cartridge, and (iii) the 27-µm screen on the pre-filter was removed to minimise retention of microbes adhering to suspended solids. Three weeks of testing in the laboratory confirmed that the placebo removed no bacteria, viruses and protozoan cysts from test water. Despite the challenge in blinding household filters, we determined after piloting that blinding the intervention would be feasible in our study area because the water had low turbidity, ranging from <5 nephelometric turbidity units (NTU) for most of the year to 10 NTU during heavy rains.

### Enrolment, baseline survey, randomization and filter deployment

After discussing the proposed study with community leaders and obtaining consent from the heads of households, a baseline survey was undertaken in April 2008 to collect information on demographics, socio-economic characteristics, and water, hygiene and sanitation practices. Data collection tools were translated in Tshiluba, the local language, and piloted before use. Following the baseline survey, households were randomly assigned to one of the two groups using a random number generator. Randomisation was stratified by village and was conducted by the trial manager who played no part in the collection of the data. Both the intervention and the placebo were distributed door-to-door by five trained field workers who were unaware of whether the device was an active filter or a placebo. Householders were trained on use and maintenance of the device according to the manufacturer's instructions. They were advised to drink filtered water directly from the tap and not to store filtered water in order to prevent recontamination. The start of follow-up period was delayed by two months due to initial technical problems with the filters.

### Blinding

The allocation sequence was concealed from both field investigators and the study population. In order to blind the intervention among assessors, field workers were divided into two teams. The team responsible for assessing health outcomes was neither involved in the distribution of the filters at the commencement of the trial nor in the assessment of the filter performance and use during follow-up. Any questions from the householders that were related to the filter were referred to and dealt with by the filter assessment team.

### Outcome Assessment

#### Diarrhoea

Investigators interviewed the female head of household or primary care giver of young children once each month over a 12-month period. They recorded any diarrhoea cases in the preceding seven days. Diarrhoea was defined as three or more loose stools passed within a 24-hour period. In an effort to further obscure the outcome of interest from the target population, field assessors also inquired about and recorded presence of fever and cough within the past seven days. Children with diarrhoea were given oral rehydration sachets and instructions on how to use them. When necessary, they were referred to the closest community health post to receive medical care free of charge. Fever and cough were also treated among young children.

#### Filter monitoring

Each month, a random sample of 30 filters and 30 placebos (25% of the total number distributed) was monitored. At each household visit, field workers noted the location and condition of the filter and recorded if the respondent was able to use and clean the filter correctly. Filter components found to be damaged were replaced. Flow rate was monitored by filling the top container with 2.5 L of water, opening the tap and measuring the time necessary to fill a 125 ml container with water. The flow rate was expressed in ml per minute.

#### Water quality

Influent and effluent paired water samples were collected for each of the selected devices. If the respondent mentioned storing the water once filtered, a third sample was collected from the container designated as the treated water storage vessel. All samples were collected in sterile 125 ml Nalgene sampling bottles and assessed for thermotolerant coliforms (TTC) within 4 h after collection. Microbiological assessment was performed using the membrane filtration technique (APHA *Standard* *Methods*) on membrane lauryl sulphate medium (Oxoid Limited, Basingstoke, Hampshire, UK) using a DelAgua field incubator (Robens Institute, University of Surrey, Guilford, Surrey, UK). Microbiological performance of the filters was expressed in terms of log reduction value (LRV) calculated as the log of the influent concentration divided by the effluent concentration (log_10_ influent/effluent).

#### Compliance

Cross-sectional surveys were conducted among each household eight and fourteen months after distribution. Participants were classified as current users if they reported using the filter ‘today or yesterday’ and if the field investigator found the filter hung for use with water in the top vessel of the device. Consistency of use was estimated by asking the respondent if he/she had drunk unfiltered water within the previous day. The survey covered further aspects on use and acceptability.

#### Blinding assessment

Immediately following the conclusion of the follow-up period, we assessed the effectiveness of blinding among participants. Blinding indices were calculated using methods developed by James and colleagues [24]and Bang and colleagues[25] Female heads of household or primary care giver were asked to identify which device they had received. Surveys were targeted at the respondents to the health surveys because they would be most likely to be influenced by their belief in treatment assignment.

### Data analysis

The analysis of the primary outcome was on an intention-to-treat basis. We used Poisson regression with robust standard errors to estimate the effect of the intervention on the longitudinal prevalence of diarrhea and other health outcomes[26]. We used generalized estimating equations (GEE) to account for clustering at the household level. Categorical data were compared using a Chi square or a Fisher's exact test where appropriate. Continuous variables were compared with a Student *t* test. Statistical analyses of microbiological data were conducted after log_10_ transformation of TTC counts to normalize the distribution. Data analysis was conducted in Stata (Stata Corporation, College Station, Texas, US).

### Ethics

The study was reviewed and approved by the ethics committee at the London School of Hygiene and Tropical Medicine and the ethics committee at the School of Public Health in Kinshasa. Written consent to participate in the research was obtained from community leaders and the head of each participating household. Investigators explained that half of the study population would be receiving effective microbiological purifiers while the others would receive placebos and that householders should continue their existing water management practices since their device may not be protective against microbial contamination. At the conclusion of the follow-up period, all placebo filters were replaced by effective filters. Following the completion of the study, the results were communicated to all study participants.

## Results

### Participant flow

**Figure 1:**
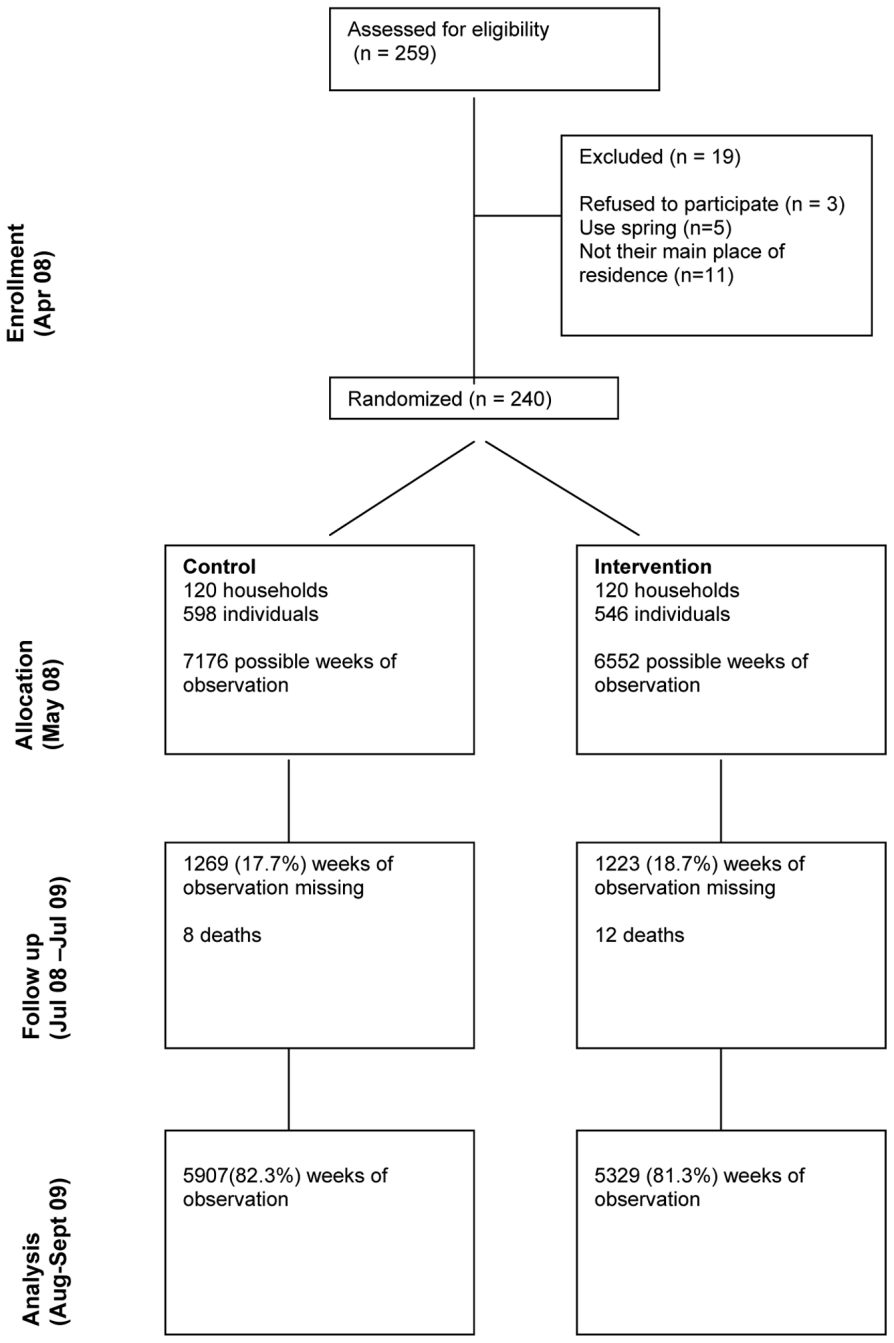
CONSORT diagram showing the flow of participants through the trial.

259 households initially volunteered to participate in the study. Nineteen households were excluded because they did not reside in the selected villages; they relied primarily on spring water for drinking, or subsequently elected not to participate. A total of 240 households were enrolled; 120 were assigned to receive the Lifestraw Family filter and 120 the placebo. Over the 12-month follow-up period, data were collected for 11,236 (81.8%) possible person-weeks of observation. Data were missing for 2492 weeks (18.2%) due primarily to participants leaving the study area or being absent at the time of visit (Figure 1). Over the study period, twenty participants died, six of them were children under the age of five. The number of deaths was 12 in the intervention group and 8 in the control group (p = 0.27).

### Baseline surveys

Intervention and control groups were similar in terms of demographic and socio-economic characteristics and hygiene and sanitation practices (Table 1). Almost all households primarily used river water for drinking. However, intervention households were more likely to store their water in clay pots and access it by dipping a cup into the container compared with control households who often used jerrycans. Only four households reported treating their water sometimes or rarely by boiling or adding bleach. Only 37% of households had a latrine and 51% had soap present in the house at the time of visit (Table 1).

**Table 1:** Baseline characteristics of participating households.

	Control		Intervention		Total	
	N	%	N	%	N	%
**Demographic and socio-economic**						
Number of households	120	(50)	120	(50)	240	(100)
Number of persons	598	(52.3)	546	(47.7)	1144	(100)
Number of households with children <5	66	(55)	57	(47.5)	123	(51.2)
Number of children <5	105	(17.6)	85	(15.8)	190	(16.6)
Mean number of persons per household	5.0		4.5		4.8	
Mean number of rooms in the house	2.2		2.3		2.3	
Respondent is female	76	(63.3)	76	(63.3)	152	(63.3)
Mean age of respondent	37.5		40.8		39.1	
Level of education						
No formal education	47	(39.2)	38	(31.7)	85	(35.4)
Primary	44	(60.3)	45	(54.9)	89	(57.4)
Secondary	29	(39.7)	36	(43.9)	65	(41.9)
Higher	0	(0)	1	(1.2)	1	(0.6)
Owns						
House	113	(94.2)	116	(96.7)	229	(95.4)
Land	115	(95.8)	117	(97.5)	232	(96.7)
Livestock	59	(49.2)	64	(53.8)	123	(51.5)
Radio	27	(22.7)	34	(28.3)	61	(25.5)
Phone	10	(8.3)	16	(13.3)	26	(10.8)
Bicycle	18	(15)	16	(13.3)	34	(14.2)
**Hygiene and sanitation**						
Use soap to wash hands	54	(45)	54	(45)	108	(45)
Presence of soap at the time of visit	65	(54.2)	59	(49.2)	124	(51.7)
Received hygiene advice in past 6 months	4	(3.4)	10	(8.4)	14	(5.9)
Presence of latrine	47	(39.2)	41	(34.2)	88	(36.7)
**Water handling practices**						
Primary source of drinking water						
River	120	(100)	117	(97.5)	237	(98.7)
Rainwater	44	(36.7)	46	(38.3)	90	(37.5)
Spring	15	(12.5)	19	(15.8)	34	(14.2)
Type of drinking water container						
Clay pot	68	(56.7)	83	(69.2)	151	(62.9)
Jerry can	50	(41.7)	30	(25)	80	(33.3)
Other	2	(1.7)	7	(5.8)	9	(3.7)
Vessel opening						
Wide mouth	71	(59.2)	92	(76.7)	163	(67.9)
Narrow mouth	49	(40.8)	28	(23.3)	77	(32.1)
Storage vessels covered	111	(93.3)	113	(95.0)	224	(94.1)
Means of obtaining water						
Pour	48	(41.0)	27	(23.3)	75	(32.2)
Dip	69	(59.0)	89	(76.7)	158	(67.8)
Treat water*	3	(2.5)	1	(0.8)	4	(1.7)

*Treat water sometimes (n = 1) or rarely (n = 3). Treatment methods boil (n = 2), bleach (n = 1), water settle (n = 1).

### Diarrhoea surveillance

**Figure 2:**
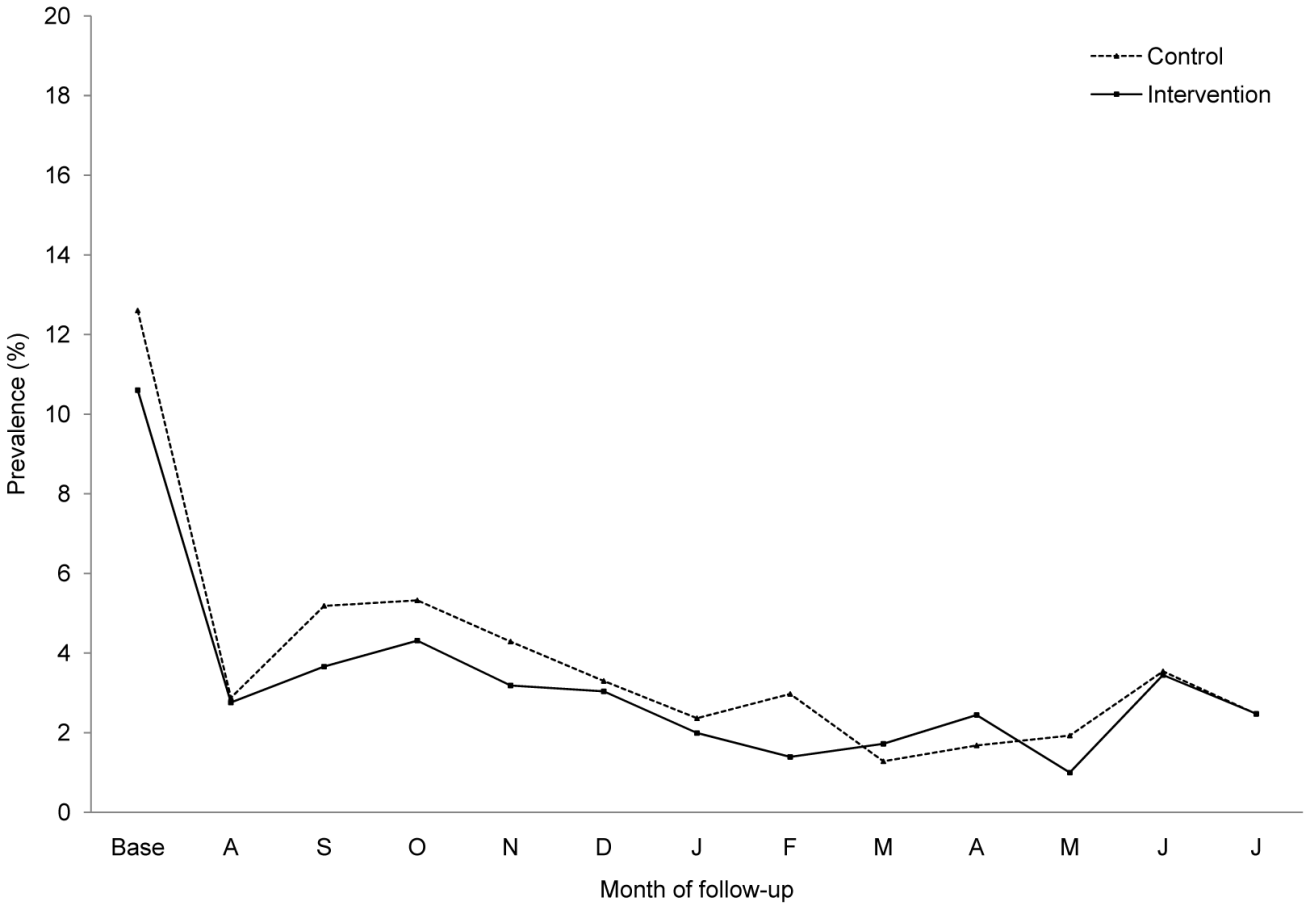
Prevalence of diarrhoea over the course of the study among participants of all ages.

At baseline, the prevalence of diarrhoea was similar in both groups (12.6% versus 10.6% for control and intervention groups, respectively). Over the 12-month follow-up period, participants of all ages who received the active filter experienced 15% fewer weeks with diarrhoea compared to those who received a placebo (mean, 2.66 versus 3.15, respectively). However, the confidence interval of the longitudinal prevalence ratio (LPR) adjusted for clustering within the household (LPR 0.85, 95% CI 0.61; 1.20) was wide and included 1. The longitudinal prevalence ratio among children under five was 0.85 (95%CI 0.56; 1.28). Figure 2 shows the prevalence of diarrhoea between intervention and control over time. We observed no difference in the mean longitudinal prevalence of fever (LPR 0.99; 95% CI 0.80; 1.22) or cough (LPR 0.99; 95% CI 0.81; 1.22) between the two groups. Health outcome data are presented in Table 2.

**Table 2:** Longitudinal prevalence of diarrhoea and other health conditions by age and treatment group.

	Mean longitudinal prevalence						LPR (95% CI)	LPR* (95%CI)
	Control			Intervention				
	Weeks of illness	Person-weeks of observation	% Weeks ill	Weeks of illness	Person-weeks of observation	% Weeks ill		
**Diarrhoea**								
<5	96	1072	**8.96**	60	801	**7.49**	0.84 (0.61; 1.14)	0.85 (0.56; 1.28)
5–15	31	1880	**1.65**	29	1765	**1.64**	1.00 (0.60; 1.65)	0.91 (0.49; 1.67)
>15	59	2945	**2.00**	52	2752	**1.89**	0.94 (0.65; 1.36)	0.95 (0.61; 1.57)
All ages**	186	5907	**3.15**	142	5329	**2.66**	0.85 (0.68; 1.05)	0.85 (0.61; 1.20)
**Fever**								
<5	249	1072	**23.23**	187	801	**23.35**	1.00 (0.85; 1.19)	1.02 (0.79; 1.30)
5–15	99	1880	**5.27**	123	1765	**6.97**	1.32 (1.02; 1.71)	1.28 (0.89; 1.85)
>15	226	2945	**7.67**	188	2752	**6.83**	0.89 (0.74; 1.07)	0.91 (0.68; 1.22)
All ages**	576	5907	**9.75**	500	5329	**9.38**	0.96 (0.86; 1.08)	0.99 (0.80; 1.22)
**Cough**								
<5	196	1072	**18.28**	162	801	**20.22**	1.11 (0.92; 1.33)	1.11 (0.85; 1.43)
5–15	163	1880	**8.67**	142	1765	**8.05**	0.93 (0.75; 1.50)	0.89 (0.63; 1.27)
>15	192	2945	**6.52**	201	2752	**7.30**	1.12 (0.93; 1.35)	1.07 (0.82; 1.39)
All ages**	551	5907	**9.33**	505	5329	**9.48**	1.01 (0.90; 1.14)	0.99 (0.81; 1.22)

*Adjusted for clustering within household.

**Age missing for 3 participants.

### Water quality

**Figure 3:**
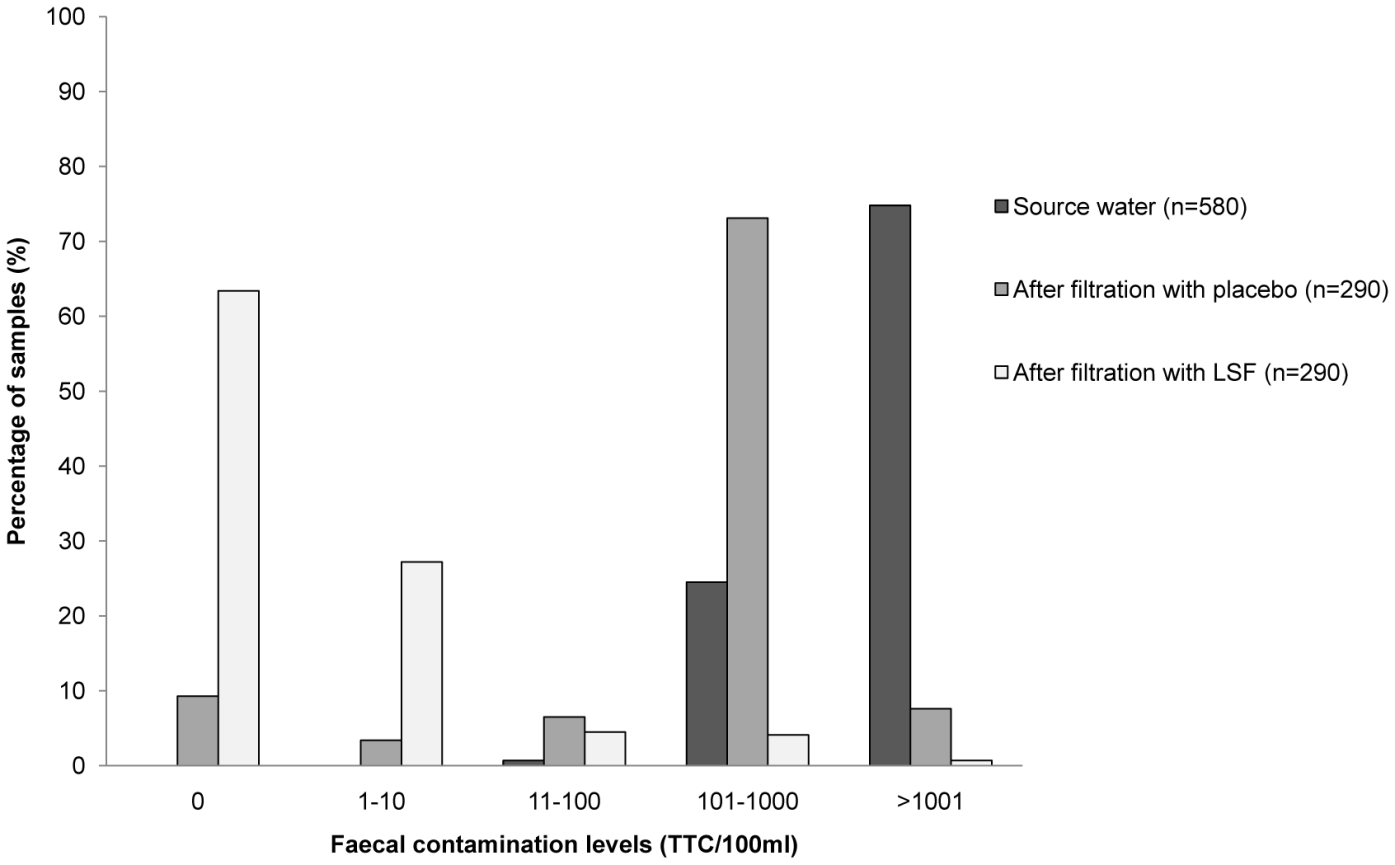
Percentage of water samples by level of contamination (TTC/100 ml).

Each device was tested on average 3 times during follow-up. 580 (81%) of the total possible paired water samples were collected. Missing samples are due to householders being absent or not being in possession of their filter at the time of visit. Source drinking water was highly contaminated, with 75% of household samples showing contamination levels above 1000 TTC/100 ml (Figure 3). The active filter achieved a LRV of 2.98 (95% CI 2.88, 3.08), removing about 99.8% of the indicator bacteria. Overall, 64% of water samples treated with filter were free of TTC and 27% had TTC levels between 1–10 TTC/100 ml. None of the filters produced water with >100 TTC/100 ml consistently over the three visits. Samples from placebos were also contaminated, with 73% of the water samples containing between 100–1000 TTC/100 ml. However, unlike the results from laboratory testing that showed the placebo to be microbiologically ineffective, results from the field showed that the placebo actually removed more than 90% of the TTC from source water (LRV 1.05, 95% CI: 0.93, 1.16).

### Flow rate

The mean flow rate of the filters over the study period was 202 ml/min (95% CI 198, 206) or 12 L/hour. It declined slightly over time (−1.5 ml/min per month, p<0.002).

### Operation and maintenance; acceptability

Over half of the respondents (56%) correctly demonstrated how to clean the filters. The pre-filter was cleaned at each use (40%) or once a day (41%), wheareas the cartrige was generally backwashed once a day (67%). Overall, 36 (12.4%) of the 290 active filters tested were found damaged during visits, mainly due to rodents chewing on the soft hoses (n = 35). Intervention households reported liking the filter due to improved aesthetics (88%), taste (92%), odour (56%) and health (35%). Reasons for dissatisfaction were slow flow rate (87%), small size of the top container (85%) and problems with rats (44%).

### Compliance

Eight months after distribution, 183 (76%) of the households were present at the time of visit and were still in possession of their filter. 68% of respondents in the intervention group could be defined as current users against 48% in the placebo group (p<0.001). However, nearly all adults (83%) and young children (95%) also reported drinking untreated water in the previous day. Fourteen months after distribution, the proportion of current users was slightly higher in both groups (76% versus 69% among intervention and control groups, respectively). Additional details about use are included in Table 3. Subgroup analysis showed no evidence of an association between use and diarrhoea morbidity (Table 4).

**Table 3:** Description of use among study participants.

	Control		Intervention		Total	
	n	%	n	%	n	%
MONTH 8						
**Last use (n = 183)** *						
Previous day	44	(48.3)	63	(68.5)	107	(58.5)
Previous week	30	(33.0)	14	(15.2)	44	(24.0)
>1 week ago	17	(18.7)	15	(16.3)	32	(17.5)
**Consistency of use on previous day (n = 107)**						
Respondent drank unfiltered water	43	(97.7)	46	(73.0)	89	(83.2)
Children (<5) drank unfiltered water	31	(93.9)	39	(95.1)	70	(94.6)
Filter accessible to young children	1	(2.3)	6	(9.5)	7	(6.5)
Store filtered water for young children	4	(12.9)	8	(19.5)	12	(16.7)
**Additional details on use in previous day (n = 107)**						
Respondent drank unfiltered water when						
In the field	33	(76.7)	39	(78.3)	72	(77.9)
In a hurry to drink	30	(69.8)	33	(71.7)	63	(70.8)
Away from village	16	(37.2)	15	(32.6)	31	(34.8)
Other	3	(7.0)	12	(26)	15	(16.8)
Children drank unfiltered water when						
Person operating the filter not present	21	(67.7)	31	(79.5)	52	(74.3)
In a hurry to drink	11	(35.5)	23	(59.0)	34	(48.6)
Away from home	10	(32.3)	13	(33.3)	23	(32.9)
Other	5	(16.1)	7	(17.9)	12	(17.1)
Did not store filtered water for children:						
No container	17	(68)	28	(87.1)	45	(78.9)
Lock the door	6	(24)	3	(9.3)	9	(15.8)
Don't want to always filter, too slow	2	(8)	0	(0)	2	(3.5)
Told not to store water	0	(0)	1	(3.1)	1	(1.7)
MONTH 14						
**Last use (n = 190)** **						
Previous day	63	(69.2)	75	(75.8)	138	(72.6)
Previous week	14	(15.4)	11	(11.1)	25	(13.2)
>1 week ago	14	(15.4)	13	(13.3)	27	(14.2)

*197 (82%) households present at the time of visit; 183 (93%) of them were still in possession of the filter and ever used it.

**203 (85%) households present at the time of visit; 192 (94%) of them were still in possession of the filter and ever used it + answer missing for 2 households.

**Table 4:** Longitudinal prevalence of diarrhoea stratified by reported last time of use.

	Mean longitudinal prevalence of diarrhoea						LPR (95% CI)
	Control			Intervention			
	Weeks of illness	Person-weeks of observation	% weeks ill	Weeks of illness	person-weeks of observation	% weeks ill	
**8 months**							
User	71	2475	**2.87**	74	3155	**2.35**	0.82 (0.59; 1.13)
Non-user	68	2420	**2.81**	41	1319	**3.11**	1.11 (0.75; 1.62)
**14 months**							
User	102	3463	**2.95**	99	3894	**2.54**	0.86 (0.66; 1.10)
Non-user	49	1642	**2.98**	27	1025	**2.63**	0.88 (0.55; 1.40)

### Blinding status

Table 5 shows respondent guesses for each treatment assignment groups. James' method, similar to the kappa statistics, produced a blinding index (BI) score of 0.42 (95%CI 0.38; 0.46). A score of 0 means that all respondents guessed correctly, 1 indicates that all respondents guessed incorrectly and 0.5 indicates random guessing. Bang's method calculates the proportion of correct guesses beyond chance in each treatment group. Bang's BI was 0.96 (95%CI 0.90; 0.99) for the intervention group and −0.63 (95%CI −0.73; −0.53) for the placebo-controlled group. Bang's blinding index varies from −1 to 1. 1 indicates complete lack of blinding, −1 opposite guess about treatment assignment and 0 random guessing. Subgroup analysis showed no evidence of an association between diarrhoea and respondents' guesses.

**Table 5:** Blinding status of respondents by group assignment at the end of the study.

	Group assignment					
Guess	Placebo*		Lifestraw Family*		Total*	
Placebo	17	(18.3)	2	(2.0)	19	(9.9)
Lifestraw Family	74	(79.6)	97	(98.0)	171	(89.1)
Don't know	2	(2.1)	0	(0)	2	(1.0)
Total**	93	(100.0)	99	(100.0)	192	(100.0)

*N (%) - number of respondents and percentage in each group.

**192 (80%) households present at the time of interview and still in possession of the filter.

## Discussion

We undertook the first double-blinded, placebo-controlled trial of household-based water filters in a low-income setting with water known to be contaminated with faecal pathogens. This design sought to assess the impact of the intervention in the absence of respondents' bias that is common in open trials. Due to challenges of developing a placebo in such settings and to successfully blinding the intervention, we monitored placebo performance and conducted a post-intervention assessment of blinding among study participants. Filter performance and health impact were monitored for a full year to account for seasonal variations and minimise the potential for exaggerated health impact often associated with shorter-term trials [9].

After adjusting for clustering, members of intervention households had 15% fewer weeks of diarrhoea than those of control households, but the confidence intervals indicated little statistical support (longitudinal prevalence ratio 0.85, 95%CI: 0.61 to 1.20). With the exception of a recent study in the United States among an elderly population^17^, this finding is consistent with other placebo-controlled trials of household water treatment interventions [14], [15], [16] which found no protective effect against diarrhoea. However, as we have observed elsewhere, those studies may have had insufficient power to identify a statistically significant impact on diarrhoea[27]. Our sample size also was not sufficiently large to detect a statistically significant difference in diarrhoea of 15%. Moreover, the baseline prevalence of diarrhoea was lower than anticipated and the clustering effect due to repeated measurement and household randomisation was higher. Pos-hoc sample size calculations indicated that we would have needed a study approximately ten times larger to achieve statistical significance.

Moreover, the placebo was not microbiologically neutral, as it removed about 90% of faecal bacteria from the source water used by control households. The reasons for this apparent effectiveness are not clear. Field staff responsible for water quality testing were extensively trained and supervised throughout the study, thereby minimizing the risk of measurement errors. One of the most plausible explanations is the formation of a biofilm resulting from adhesion of suspended solid particles and bacteria on the inner surface of the plastic pipe forming the placebo cartridge. The effectiveness of the placebo rendered our trial a comparison between a 1-log filter and a 3-log filter. Studies have reported an association between 1 log removal of faecal bacteria from drinking water and a reduction in diarrhoeal disease [28], [29]. Our results may therefore understate the effectiveness of the active filter if it were compared to a true placebo. These results suggest that in this setting with relatively high levels of microbial contamination in source water, a filter of superior microbiological performance may be more effective at preventing diarrhoea than one that removes only 90% of waterborne pathogens. This finding, if validated in future studies, would support the need for high performance standards in water treatment devices in order to optimize health benefits.

The blinding of the intervention was not successful. In both treatment groups, the vast majority of survey respondents believed that they had received the active filter, although this proportion was significantly lower in the placebo group. Unsuccessful blinding means that we cannot rule out the possibility that the observed effect on diarrhoea is unbiased. However, the interpretation of blinding indices is not always clear[30]. The fact that a large proportion of control households remained blinded throughout the trial suggests that respondents' bias may have at least been partly reduced. The smaller effect size we observed here may be indicative of a less biased estimate compared with open trials. Our estimate is similar to the pooled estimate of effect of open trials of ceramic filters after adjustment for lack of blinding[13]. The fact that ‘control’ health conditions (fever and cough) remained unchanged by the intervention also suggests that blinding may have been effective, although the usefulness of this approach to detect the presence of respondents' bias has not been validated. Including a third arm with no intervention would have provided a better understanding of the role of bias in this study.

Under field conditions, the Lifestraw Family filters were effective in removing faecal bacteria from source water. Two-thirds of filtered water samples were free of faecal coliforms while most of the remaining samples had low levels of contamination. The fact that specific filters did not consistently produce contaminated water suggests that contamination may have occurred during collection of the sample, perhaps from the tap. The flow rate was higher than that observed in laboratory conditions, possibly due to lower water turbidity at the study site (compared to lab testing at 15 NTU) or inconsistent use by householders. The damage rate was high although the most common problems were due to rats eating the soft plastic components.

Eight months after distribution, two-thirds of the respondents met the study's definition of current users, although almost none of them drank filtered water exclusively. This pattern of use was seen among both adults and children under five. Participants drank unfiltered water when spending time outside their home, but also when they felt eager to drink and did not want to wait for filtration. Young children did not have access to the filter when their parents were away from home. In accordance with the manufacturer's instructions, householders were advised to use water directly from the filter and not to store treated water due to the risk of recontamination. Consistent with these instructions, almost none of the households stored filtered water for their children, though many lacked a storage container even if they had chosen to do so. The manufacturer has advised that in future deployment of the filters, it will consider changes in instructions to encourage safe storage of treated water or provide a storage vessel for the filtered water to help increase exclusive consumption of treated water, especially by this vulnerable group of young children. However, there is also evidence that even occasional consumption of untreated water may eliminate the protective effect of water treatment[31] and changes to the configuration of the filter may not be sufficient to increase exclusive use unless accompanied by fundamental changes in behaviour to increase compliance.

Our study had certain additional limitations. The study sites were not randomly selected, but were chosen based on eligibility criteria that included high levels of faecal contamination in source water and high prevalence of diarrhoea at baseline. Accordingly, these results are not necessarily generalizable to other populations in the Congo or beyond. Second, the use of a seven-day recall period is known to produce less precise estimates compared with a 48-hour recall period[32].

Our study provides little evidence of a protective effect of the filter against diarrhoea. Nevertheless, an effect of 15%, which we observed but could not confirm here, would represent a substantial impact on diarrhoea, a major killer of young children. Future studies with sufficient power to detect this effect size will be necessary to determine the magnitude of any effect against a neutral placebo and to confirm that the effect is not attributable to chance. Our study also demonstrates the need to monitor placebo performance and the challenge of blinding household-based water treatment interventions under adverse conditions.

## Supporting Information

Protocol S1 (0.08 MB DOC)

Checklist S1 (0.19 MB DOC)
