# Supplementary material for: Field Assessment of a Novel Household-Based Water Filtration Device: A Randomised, Placebo-Controlled Trial in the Democratic Republic of Congo
Source: PLoS One. 2010 Sep 10;5(9):e12613. doi: 10.1371/journal.pone.0012613 (PMC2937016; doi:10.1371/journal.pone.0012613)
Supplement: Protocol S1 — (0.08 MB DOC) [file pone.0012613.s001.doc]

**A randomised controlled trial TO ASSESS**

**THE LIFESTRAW Family® household gravity filter**

***Study Protocol***

***Department of Infectious and Tropical Diseases***

***London School of Hygiene & Tropical Medicine***

**A. Background**

Diarrhoea is the second most important cause of mortality among children less than 5 in developing counties and claiming 2.5 millions lives each year (Kosek 2003). Interventions to improve water quality at the household level have been shown highly effective in reducing diarrheal diseases (Clasen 2007).

Over the past few years, the WHO has actively encouraged the development of new point-of-use water treatment technologies that are effective, acceptable and affordable to populations in low-income countries. Among the most common methods are chlorination, ceramic filtration, solar disinfection and boiling (Sobsey 2002). However, each of these has limitations. For examples, chlorination is often associated with an unpleasant taste and smell and is less effective in highly turbid water. Ceramic filters can have a slow flow rate and tend to be less effective with small enteric pathogens such as viruses.

To overcome these shortcomings, Vestergaard Fransen SA has developed a new household-based gravity filter system “LifeStraw Family” that uses ultrafiltration mechanisms to remove pathogens. The filtration unit consists of a hollow-fibre cartridge of 20 nm diameter pore size. Untreated water is poured into a 2L container, flows down a 1 m long pipe and becomes purified as it passes through the cartridge. Microbiologically safe water can be directly accessed via a tap. The filter system is designed for suspension on a wall and the length of the pipe was chosen specifically to improve flow rate. The cartridge is also equipped with a backwash inlet to facilitate cleaning of the filtering unit, thus preventing clogging and improving longevity. Independent laboratory testing of the cartridge have shown high microbiological performance, meeting the US EPA standards of 4-log reductions for viruses. The device is designed to last for about a year and to treat up to 10,000 L of water with a flow rate of approximately 6L/hour. The flow shuts down when the hollow-fibres are exhausted, indicating when to replace the cartridge.

**B. Objectives**

The objectives of the study are (i) to assess the effectiveness of the LifeStraw Family household filter in preventing diarrhoeal diseases (ii) to determine the effectiveness of the LifeStraw household filter in reducing the bacterial load of drinking water and (iii) to evaluate the extent to which it is used correctly and consistently by, and is acceptable to, the target population (iv) finally, we will measure longevity of the filter as it is used in a field setting.

**C. Methods**

***1. Trial design and setting***

A two-arm randomised placebo-controlled trial will be conducted over a 12-month period. The trial will include two groups of households: one group receiving the LifeStraw Family filter and the other group receiving a placebo version of the LifeStraw filter. The study will be conducted in a rural setting with poor water supply facilities and the community will be selected based on the following criteria (i) reliance on highly contaminated drinking water sources including unprotected open wells, and surface water sources with contamination levels higher than 1000 TTC/100ml (ii) Low use of household water treatment methods such as boiling, filtration etc. (iii) A high representation of children under 5 in the population (iv) Strong willingness from community members and leaders to take part in the project. The site will be selected following a preliminary site assessment visit during which samples will be taken from water sources and analysed.

***2. Enrolment and randomisation***

Households who are willing to take part of the study are eligible if they don’t treat their water in a way that is deemed effective to prevent diarrhoea (e.g. boiling, filtration with a state of the art filter, regular use of bleach). After being given full details of the study, the head of household will be asked to consent in writing to participate. A copy of the information and consent form is attached as Appendix A. Prior to the commencement of the study; a baseline survey will be conducted. The head of household will be interviewed about family demographics, household possessions (as a measure of estimated wealth), water source, and water handling and purification practices. The baseline questionnaire will be translated into the local language and piloted before use. Following baseline, household participants will be randomly assigned to one of the two groups by using a list of consecutively numbered households and a random number generator or by conducting a lottery. Randomisation will be conducted by a member of staff not involved in the data collection. The study participants and the field investigators will be blinded to group assignment.

***3. Study intervention***

Half of the participating households will receive the Lifestraw filter and the other half will receive a filter which looks like the Lifestraw filter but which does not remove pathogens from the water.

The placebo has the same outside components as the real filter but the filtering membranes inside the cartridge have been replaced by some extra piping to imitate the weight of the real cartridge and the flow coming out of it. The 27-micron layer on the pre-filter has been removed to ensure that the placebo does not retain any bacteria. The placebo device has been tested in the laboratory and was shown to be ineffective in removing pathogens from the water.

Households will be trained on how to use their filter according to the manufacturer’s instructions. They will also be advised to continue following their usual drinking water practices so that the use of the placebo is not increasing their risk of drinking contaminated water. No hygiene instructions will be provided to study participants.

***4. Outcome assessment***

Diarrhoea prevalence

Within one month of distributing the filters, field investigators will visit each household once a month (i.e. 12 visits per household during the overall follow-up period) to conduct a health survey. They will record for each household member, whether she/he had experienced any illness in the past 7 days, prompting a list of symptoms - in which diarrhoea is included - if no answer was spontaneously given. Repeated point prevalence has been shown as more valid and less resource-intensive method for estimating longitudinal prevalence compared with continuous surveillance (Schmidt, 2007). Formative research will be conducted prior to the intervention in order to explore local perception of diseases. Based on these findings, diarrhoea will be self-defined (Ross, 1994) or defined according to the standard WHO definition of 3 or more loose stools in a 24 hr period. Any medical conditions observed by field staff during visits will be referred to the local medical centre.

Microbial water quality, filter use and longevity

In each of the two groups, a random sample of households (25%) will be selected every month to assess microbiological performance of the filters. Field staff will conduct unannounced random visits and will take two samples: one sample of untreated drinking water before it is poured into the filter and one from filtered water coming out of the system’s tap. If the household store the filtered water for drinking, a third sample will be taken from the container in which filtered water is stored. During the visit, field investigators will also fill a brief observations checklist on the filter’s appearance and potential problems encountered with the filter. If the filter is no longer in use, the reason given by the user will be recorded. If the filter has clogged, the time at which the filter went out of service will be noted and the filter will be replaced by a new one. This will be used to assess longevity. All samples for microbiological analysis will be collected in sterile Whirl-Pack™ Bags. Samples will be placed on ice and processed within 4 hours of collection to assess levels of thermotolerant (faecal) coliforms. Microbiological assessment will be performed using the Oxfam-DelAgua Portable Water Testing Kit in accordance with the kit’s instructions.

Acceptability

Acceptability of the intervention will be evaluated through questionnaires administered at 4, 8 and 12 months of follow-up. A random sample of the female heads of households will be asked to participate in focus group discussions. All acceptability material such as questionnaires and focus group discussion guide will be piloted before use.

Blinding

Field investigators responsible for conducting health surveys will be different from those involved in filter distribution, filter inspections and water sampling and analyses. During the follow-up, a survey will be conducted among both participants and health surveyors in order to evaluate whether the intervention was successfully blinded.

**5. Communication of results to community members**

Following the completion of the study, the results of the disease surveillance will be communicated to all study participants at a meeting called for that purpose. If the intervention has been effective in reducing disease, households who received the placebo at the beginning of the study, will be given the effective filter.

**6. Data analysis**

Data will be recorded on an Excel spreadsheet and analysed in Stata software (version 9). The primary analysis will consist of comparing longitudinal prevalence of diarrhoea between the intervention and the control groups.

Secondary analyses include:

- Comparison of faecal coliforms (TTC per 100ml) in the water before and after filtration.
- Assessment of longevity of the filters
- Descriptive analysis of use and acceptability data

Diarrhoea prevalence will be analysed using appropriate statistical procedures which adjust for clustering within household and for repeated measures over time. Multivariate analysis will adjust for potential confounders (e.g. age, level of wealth etc). The quantitative outcomes will be analysed with appropriate parametric and non-parametric methods.

Sample size

The sample size required for meeting the primary objective of the study i.e. detecting a 30% reduction in diarrhoea prevalence among individuals from the intervention group compared to individuals from the control group was calculated based on the following assumptions: a significance level of 0.05 (two-sided), 80% power, a baseline prevalence of diarrhoea of 5%, a coefficient of variation of 2. The following formula was used:

**N** = [(0.84+1.96)^2^ x (sd_0_^2^+sd_1_^2^)] / (p_0_-p_1_)^2^ = **511** Individuals

Where:

- sd_0_ and sd_1_ are the standard deviation among the control group and intervention group respectively;
- p_0_ and p_1_ are the expected diarrhoea prevalences among control and intervention group.
- Standard deviations were obtained as following: sd_0_=0.05*2=0.1 and sd_1_=0.035*2=0.07.

In order to account for lost to follow-up (10%) and clustering of diarrhoea within household, the sample size will be 600 individuals in each arm. Assuming 5 persons per household on average, the number of households to be recruited in each arm is 120 households per arm so approximately 240 households in total.

**D. REFERENCES**

Clasen T, Schmidt WP, Rabie T, Roberts I, Cairncross S (2007). Interventions to improve water quality for preventing diarrhoea: systematic review and meta-analysis. British Medical Journal, 334:782.

Kosek, M., Bern, C., Guerrant, R.L. (2003) The global burden of diarrhoeal disease, as estimated from studies published between 1992 and 2000. Bulletin of the World Health Organisation, 81: 197-204.

Schmidt WP, Luby S, Genser B, Barreto M, Clasen T (2007). Estimating the longitudinal prevalence of diarrhea: an alternative to continuous surveillance. Epidemiology, 18:537-43.

Ross DA, Huttly SA, Dollimore N, Binka FN (1994). **Measurement of the Frequency and Severity of Childhood Acute Respiratory Infections through Household Surveys in Northern Ghana** International Journal of Epidemiology; 23: 608-616.

WHO (2004). Guidelines for *Drinking-* *Water Quality*, Vol. 1. Third Edition. World Health Organization, Geneva, Switzerland.

WHO (2007). Combating waterborne disease at the household level. World Health

Organization, Geneva. Available from <http://www.who.int/household_water/advocacy/combating disease/en/index.html>
